# Color and Grey-Level Co-Occurrence Matrix Analysis for Predicting Sensory and Biochemical Traits in Sweet Potato and Potato

**DOI:** 10.1155/2024/1350090

**Published:** 2024-10-30

**Authors:** Judith Ssali Nantongo, Edwin Serunkuma, Gabriela Burgos, Mariam Nakitto, Joseph Kitalikyawe, Thiago Mendes, Fabrice Davrieux, Reuben Ssali

**Affiliations:** ^1^International Potato Center, Ntinda II Road, Plot 47 PO Box 22274, Kampala, Uganda; ^2^International Potato Center, Lima, Uganda; ^3^International Potato Center, Nairobi, Kenya; ^4^CIRAD, UMR Qualisud, Montpellier F-34398, France

**Keywords:** breeding, digital agriculture, high-throughput phenotyping, machine learning

## Abstract

In sweet potato and potato, sensory traits are critical for acceptance by consumers, growers, and traders, hence underpinning the success or failure of a new cultivar. A quick analytical method for the sensory traits could expedite the selection process in breeding programs. In this paper, the relationship between sensory panel and instrumental color plus texture features was evaluated. Results have shown a high correlation between the sensory panel and instrumental color in both sweet potato (up to *r* = 0.84) and potato (*r* > 0.78), implying that imaging is a potential alternative to the sensory panel for color scoring. High correlations between sensory panel aroma and flavor with instrumental color were detected (up to *r* = 0.66), although the validity of these correlations needs to be tested. With instrumental color and texture parameters as predictors, low to moderate accuracy was detected in the machine learning models developed to predict sensory panel traits. Overall, the performance of the eXtreme Gradient Boosting (XGboost) was comparable to the radial-based support vector machine (NL-SVM) algorithm, and these could be used for the initial selection of genotypes for aromas and flavors (*r*^2^ = 0.64–0.72) and texture attributes like moisture or mealiness (*r*^2^ > 50). Among the chemical properties screened in sweet potato, only starch showed a moderate correlation with sensory features like mealiness (*r* = 0.54) and instrumental color (*r* = 0.65). From the results, we can conclude that the instrumental scores of color are equivalent to those scored by the sensory panel, and the former could be adopted for quick analysis. Further investigations may be required to understand the association between color and aroma or flavor.

## 1. Introduction

Texture and color are among the most desirable traits for consumers of sweet potato and potato [1] and can provide essential information for image processing and pattern recognition. Biochemical composition is the base for color and texture [2]. Machine learning and artificial intelligence are among the latest emerging trends in the computer field that could have their application in image processing. These have been used in healthcare, cybercrime, biochemistry, robotics, metrology, banking, medicine, marketing, and consumer research, among others, to solve complex research problems [3, 4]. In classical plant breeding studies, computer vision, machine learning, and deep learning aspects of artificial intelligence have been successfully integrated into noninvasive phenotyping techniques such as near-infrared (NIR) spectroscopy and imaging [5–7], that is gradually improving the efficiency of data collection and analysis. In machine learning, neural networks, partial least squares regression (PLS), random forest (RF), and support vector machines are some of the methods that have been widely applied to analyze high-throughput phenotyped, nonlinear, and complex data [5, 8–10]. Since phenotyping speed is among the major limitations to accelerated breeding, these statistical and computer developments to accelerate phenotyping should enhance the development and deployment of superior genotypes.

In sweet potatoes and potatoes, high-throughput phenotyping using noninvasive imaging techniques has considerably progressed. Researchers have used digital imaging [6], NIR spectroscopy [11, 12], thermal imaging [13], and chlorophyll fluorescence [14] to evaluate root or tuber physical properties, sensory traits, chemical components, varietal authentication, and defect aspects, among others. Digital imaging, using visible and other wavelengths of light, is the lowest-costing and easiest-to-use imaging technique. It has also become more common following the development of high-precision cameras in mobile phones and other laboratory imaging instruments, for example, the DigiEye. Moreover, acquiring images is less laborious and may reduce subjectivity and, sometimes, the inconsistency of human-based evaluations. Using images for evaluating traits may also enhance the transferability of models between laboratories or industries [15].

In the visible spectral range (400–700 nm), images comprise pixels commonly represented as a combination of the red, green, and blue (RGB) color channels. The pixels can be analyzed to depict variations in color, morphology, and texture within and between samples [16, 17]. Color analysis is straightforward, involving the detection of color distribution and averages within and between samples. Further analyses may also include correlating color distribution with other variables [17] such as sensory panel values and biochemical attributes as presented in this manuscript. Texture analysis involves the measurement of heterogeneity in the tonal values of pixels within a defined area of an image. Various methods are available to analyze image texture [18], although the computation of grey-level co-occurrence matrix (GLCM) [19] is popular because of its superiority, simplicity, and adaptability [20, 21]. The GLCM method considers the spatial relationship of pixels by calculating how often pairs of pixels with specific values and in a specified spatial relationship occur in an image. GLCM is a common texture analysis method, especially in the geosciences and remote sensing fields of agricultural research, but its use in predicting plant traits for breeding is still limited [10, 22]. This study aimed at quantitatively investigating the relationship between extracted image features and biochemical and sensory panel traits to enable future high-throughput characterization of the latter in the breeding process. Two specific objectives were explored: (i) determine individual color, image texture, and biochemical features that strongly correlate with sensory panel traits and, (ii) select the modeling techniques that would provide the best sensory panel prediction results, using image color and texture as predictor traits.

## 2. Methods

### 2.1. Root and Tuber Sample Collection

Sample collection and image acquisition were described in earlier documents [23]. In summary, fresh roots of 99 sweet potato samples were collected from International Potato Center (CIP) breeding trials located in six different sites shown on the map (Supporting Information Figure 1), representing different agroecological zones (AEZ) of Uganda. These were harvested during the two growing seasons of 2021. The potato samples were collected from the National Agricultural Research Organization (NARO) breeding trials in Uganda and the CIP trials in Kenya. A total of 51 samples were collected. The samples were collected in triplicate and comprised 33 and 17 different sweet potato and potato genotypes, respectively. These reflected a wide genetic and environmental diversity, suitable for modeling. 

### 2.2. Image Collection

Images were collected on raw and cooked slices as described in earlier documents [6]. In brief, the roots or tubers were washed, peeled, and cross-sectionally sliced. Images were then captured on the slice in the laboratory using the DigiProduction imaging system, also known as the DigiEye 700 mm Cube (VeriVide Limited, Leicester, United Kingdom), mounted with a D7500 Nikon DSLR camera (Nikon Cooperation, Tokyo, Japan). The DigiEye machine is a 64-bit modular computer-controlled digital imaging system that comprises an illumination cabinet, digital camera, desktop personal computer, and monitor calibration. It was calibrated with the DigiTizer (VeriVide Ltd., Leicester, United Kingdom) color chart to characterize the camera response by relating its RGB signals to CIE specifications. The cabinet is equipped with two VeriVide D65 standard illuminant emulators, which illuminate the samples consistently under stable lighting conditions [6]. Lamps were switched on at least 10 min before the measurements according to manufacturer indications to stabilize them. All images were collected in triplicate per sample. The CIELAB color space was used. For each image, a TIF file was stored.

### 2.3. Feature Extraction

**Figure 1:**
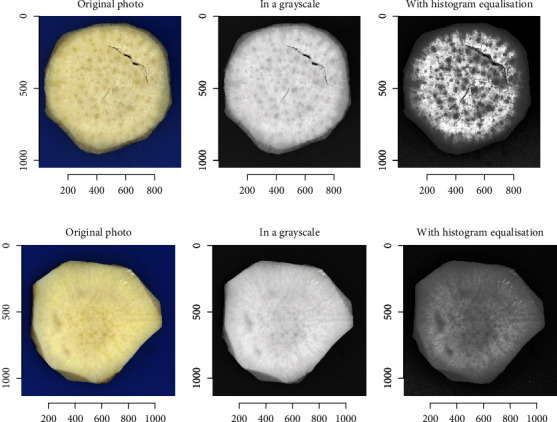
Region of interest (marked as nonblack/blue pixels) of a slice of cooked sweet potato root in original, greyscale, and with histogram equalization of (a) very mealy and (b) less mealy sample.

**Figure 2:**
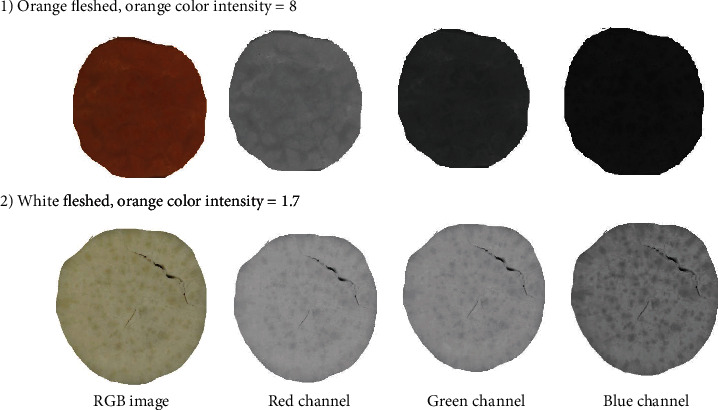
Region of interest (marked as nonblack pixels) of (1) orange and (2) white-fleshed sweet potato samples at different RGB (R = red; G = green; B = blue) color channels.

Image preprocessing was first done by removing the background. Then, color and texture statistics were extracted from the regions of interest. The LABCH color values were extracted directly from the DigiEye software, and the three channels of RGB (e.g., Figures 1 and 2) were extracted from jpeg images using the jpeg R package. GLCM statistics for texture analysis were processed from TIF images using the GLCM package in R-statistics [24]. The matrices were computed in all four directions (0°, 45°, 90°, and 135°) and then combined into one rotation-invariant texture. There was no difference in the results among the different directions (results not shown). A 9 × 9 sliding window was used and did not differ from the 3 × 3 sliding window results (not shown). Eight different GLCM features commonly found in the literature were obtained from the samples, namely, mean (the weighted average of the probability of occurrence of features based on their location on the GLCM map), variance (the spread of grey-level values in the GLCM matrix), homogeneity (the similarity in intensity levels between neighboring pixels), contrast (the local variation in the grey-level intensities), dissimilarity (the difference in intensity levels between neighboring pixels), entropy (the randomness or complexity of the texture in the image), angular second moment (the uniformity of grey-level values in an image), and correlation (the linear dependencies of greyscale values and the degree of (an) isotropy in the microcomputed tomographic images of each of the sample types) [25]. 

### 2.4. Reference Data: Descriptive Sensory Analysis and Texture Evaluation of Cooked Sweet Potato/Potato

The sensory traits evaluated in sweet potato and potato have been presented in earlier papers [7, 11, 12]. The protocol for descriptive sensory analysis established for sweet potato that was used has been previously described [26, 27]. In summary, up to 12 trained panelists evaluated steamed sweet potato and potato genotypes. Orange color intensity, pumpkin flavor, pumpkin aroma, sweet potato aroma, and sweet potato flavor on a 10-point scale range from minimum (0) to maximum (10). Similar scoring was done for potatoes. The sensory panel was first accurately trained and evaluated for appropriate discriminant ability and repeatability. All sensory data were first organized in Microsoft Excel (Microsoft 365 Apps for Enterprise, version 2102). The data from genotypes served in duplicate (sensory replicates) were used to evaluate panel reliability in SPSS version 22 (IBM Corp. in Armonk, NY, 2013) by Fisher's test with genotypes and panelists as fixed factors [27]. The mean, standard deviation (sd), minimum, and maximum values associated with the evaluated traits were calculated in Excel (Supporting Information Table 1) and were presented in earlier documents [7, 11]. All descriptors and definitions are based on the lexicon developed by [27].

### 2.5. Biochemical Data From Sweet Potato Roots

To evaluate the dry matter of sweet potato genotypes, approximately 2 g of thinly sliced raw sweet potato roots from each genotype was accurately weighed into a moisture dish in triplicate, and the dry matter was evaluated following the standard operating procedure [28]. Starch content was estimated by the complete acid hydrolysis method [29]. A flour sample of 2.5 g was suspended in a mixture of 200 mL of water and 20 mL of HCl acid. The mixture was heated in a flask provided with a reflux condenser for 2.5 h. The contents were cooled and neutralized with NaOH (5 N). The volume was made to 250 mL, and the sugar formed was determined as dextrose by the Lane and Eynon reducing sugar estimation method. The dextrose multiplied by 0.9 was taken as starch. Protein was determined according to the Association of Official Analytical Chemistry [30]. The total amylose content of sweet potato starch was determined using the spectrophotometric method after the removal of lipids from starch with hot 75% n-propanol for 7 h in a Soxhlet extractor [31]. The pure potato amylose (Sigma-Aldrich) and amylopectin from maize (Sigma–Aldrich) were used to create a standard curve, and the total amylose content of each sample was inferred from this standard curve. The moisture content of the raw and cooked sweet potato was determined by drying a 2 g sample in an oven set at 50°C for 2 weeks.

### 2.6. Statistical Analysis and Modeling

The relationship between sensory panel traits and DigiEye image features of sweet potato/potato samples was investigated using Pearson's correlation coefficient, *r*, which is normally used to measure the strength of the linear relationship between two variables. To develop regression models for predicting the sensory panel traits based on color and texture image features, machine learning-based models were used. These were linear regression (LM), PLS, principal component regression (PCR), linear and nonlinear support vector machines (L-SVM and NL-SVM), elastic net regression (ENR), extreme gradient boosting (XGBoost), RF, k-nearest neighbors algorithm (KNN), and classification and regression tree (CART) analysis. Because of the limited sample size, models were employed on training datasets only. All analyses were implemented in R (v 4.3.1).

## 3. Results and Discussion

**Figure 3:**
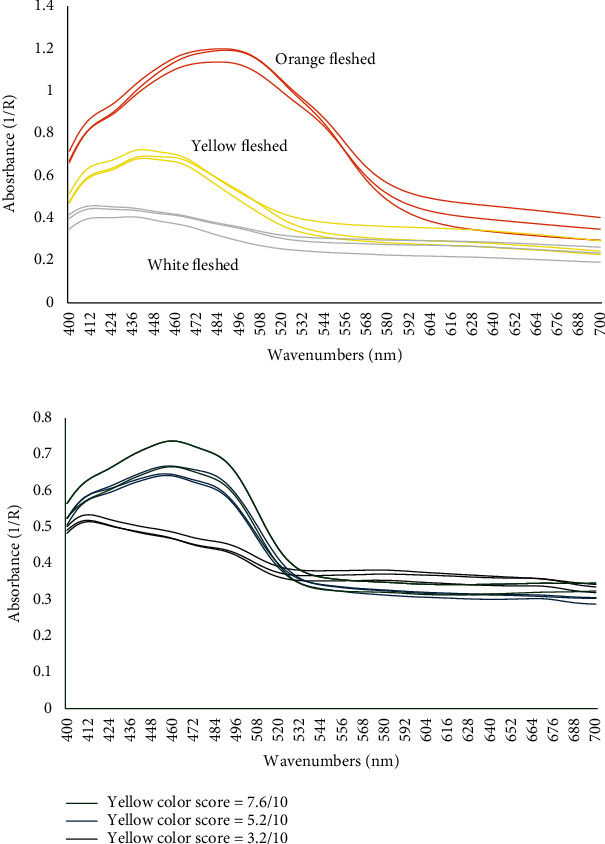
The spectral reflectance of (a) cooked sweet potato roots and (b) cooked potato tubers of different color scores.

The spectral reflectance of the sweet potato root and potato tuber in the visible region (400–700 nm) is shown in Figure 3.

The spectral reflectance curve reflects the visual characteristics of the object. All sweet potato roots and potato tubers are known to have carotenoids, which range from colorless to orange. Carotenoids have a broad absorption range in blue (400–500 nm), which suggests the peaks in Figure 3. The intensity of absorption is higher in sweet potato than potato, and higher in the orange-fleshed sweet potato reflecting the amount of carotenoids in the different genotypes. However, the carotenoids overlap with the chlorophyll spectrum.

### 3.1. Correlation Between Image Features and Sensory Traits

#### 3.1.1. Instrumental Versus Sensory Panel Color

High correlation coefficients (*r*) (Tables 1 and 2) were detected between the sensory panel color and instrumental LABCH (Tables 1 and 2) and RGB (Table 3) color channels for both cooked and raw sweet potato and potato samples.

In cooked sweet potato, on the LABCH scale, sensory color scores were strongly positively correlated with saturation (C, *r* = 0.84) but negatively correlated with hue (H, *r* = −0.84). Similarly, in potato, high correlations between sensory yellow color and instrumental color were detected (Tables 1 and 2). On the RGB channel, high correlations were detected between the sensory color scores and the instrumental green and blue channels (Table 3), since there is minimal contrast in the red channel (Figure 2). In potatoes, contrastingly, the sensory yellow color intensity correlated mostly with the red pixels (*r* > 0.78), with no correlation with the green and blue pixels. Similar trends were detected in the raw samples of sweet potato (Supporting Information Table 1), suggesting that the color of the cooked samples can be well predicted based on raw samples.

It is vital to evaluate color as it is a fundamental trait, playing a major role in quality evaluation and the decisions of the consumer [15], in addition to its relationship with plant pigments and, therefore, plant development and biochemical and nutrition changes. Studies have indicated that color can easily be measured by instrumental methods such as colorimeters or spectrophotometers, and for homogeneous opaque materials with a smooth surface, the measured and sensory colors will be similar. However, few food products are homogeneous, including sweet potatoes and potatoes. The surface texture, gloss, shape, and form of food products have a dramatic influence on the human perception of color, and hence, developing instrument-based models will reduce subjectivity. Different food products also exhibit different color ranges, which are affected by the type of food crop, the environment where it is grown, and the preparation process [32]. Overall, the sensory panel color of sweet potato and potato can be well predicted based on the instrumental measurements of the raw or cooked samples, and there is no need to develop complex prediction models for sweet potato or potato flesh color. We noted that the color correlations did not differ between the cooked and the raw samples, suggesting that the tuber and root color of the cooked products could be sufficiently predicted based on the raw samples.

#### 3.1.2. Instrumental Color Parameters Versus Other Sensory Panel Traits

Moderate correlation estimates were detected between the sensory panel aroma, taste, or flavors and the instrumental image color parameters (Table 3). In sweet potato, moderate correlations were detected between sensory panel pumpkin aroma/flavor with instrumental color values (Table 3). Interestingly, the same correlation was detected in potatoes, where the red pixels also moderately correlated with several sensory panel taste and flavor traits (*r* > 0.50). There is no clear biochemical basis for these correlations, especially when using images, and it is not certain whether they have meaningful cause-and-effect relationships. However, psychophysical studies have suggested the presence of relationships between color and flavor [33], where studies have documented the interaction of sensory-based color, flavor, aroma, and texture, and that certain food colors are involuntarily associated with specific flavors and aromas [33, 34]. Anecdotal field information has also suggested an association of certain colors and tastes or flavors in sweet potato and potato, although this hypothesis needs further testing. Interestingly, on the same samples, earlier results also strongly associated aroma with spectral information, suggesting that aroma could possibly be predicted based on spectral data [7], since spectra model the biochemical composition of samples. This could suggest that the biochemical composition responsible for color in sweet potato and potatoes could also be associated with flavor and aroma. Studies in coffee have also modeled aroma based on hyperspectral imaging [35], which emphasizes the possibility of predicting aromas in sweet potato and potatoes using imaging.

#### 3.1.3. Sensory Panel Traits and GLCM Texture Parameters

Moderate to weak correlations were detected between sensory panel parameters and GLCM texture parameters. In sweet potato, with a single feature, the best GLCM feature can only account for 0.67% variability (based on the correlation coefficient, *r*) of the sensory panel scores. The highest correlations were detected between pumpkin aroma or flavor and the mean of grey pixels (GLCM mean) (Table 4). We expected that in line with earlier computer vision studies [6], mealiness could be explained by the GLCM texture parameters since there is a visible pattern of graininess in mealy samples (Figure 1). However, this was not the case, since the best single GCLM feature accounted for only 0.51% variability (based on the correlation coefficient, *r*) of the sensory panel scores of mealiness, suggesting additional factors than a spatial arrangement of grains in the samples explain mealiness. Computer vision seems to perform well in predicting sensory traits even in other crops [36, 37].

Additionally, orange color intensity and texture-related traits such as firmness, moisture in mass, plus crumbliness were weakly correlated with GLCM texture features (*r* = 0.40–0.50). This indicates that the arrangement of the pixels at the grey level partially explains the mealiness and other texture traits. In potatoes, all the sensory texture, taste, flavor, aroma, and color attributes were also weakly correlated with GLCM texture features, where the highest correlations were detected between yellow color and GLCM homogeneity (*r* = 0.34) or 2nd-moment and contrast (*r* = 0.37). Even for color, moderate correlations were detected with GLCM features. Among instumental parameters , the highest correlations were between GLCM mean or variance and LABCH or RGB color metrics (Supporting Information Tables 2/3) in sweet potato. In potatoes, red color parameters moderately correlated with GLCM homogeneity (*r* = 0.64–0.67), entropy (*r* = 0.59–0.60), and 2nd moment (*r* = 0.50). This suggests that the red pixels strongly associate with the texture of the potatoes. However, it is possible that a combination of specific features could predict these traits better. Therefore, future studies could employ specific feature selection for the desirable traits to decide which ones could be more important.

#### 3.1.4. Biochemical and Sensory or Image Properties

In cooked sweet potato, the highest correlations among biochemical (total starch, proteins, and amylose) and sensory traits were detected between starch and mealiness (*r* = −0.54) and moisture in mass (*r* = 0.52). This suggests that high starch genotypes retain more water, which works against the mealiness of the product. Between biochemical and image traits, the amount of starch in sweet potato roots also positively correlated with L (lightness) of the LABCH color spectrum (*r* = 0.65). No meaningful correlations were detected with GLCM features. The amount of protein negatively correlated with GLCM maximum contrast (*r* = −0.60) and dissimilarity (*r* = −0.52) of grey pixels, but not with color features. The same trend was observed with raw sweet potato samples but with lower correlation coefficients (results not shown).

### 3.2. Model Development

**Figure 4:**
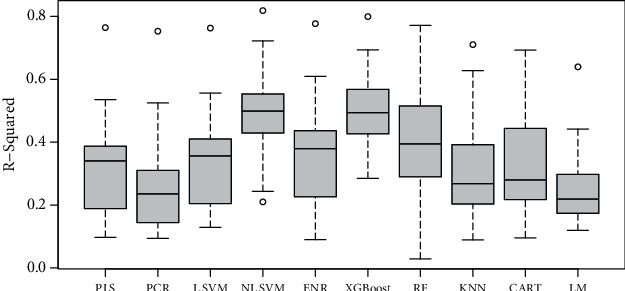
Boxplots of *r*^2^ values for different statistical models used to predict sensory traits in cooked sweet potatoes.

**Figure 5:**
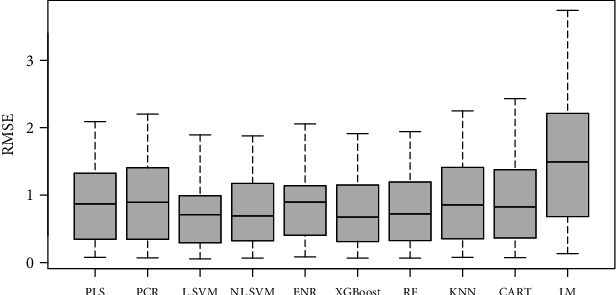
Boxplots of RMSE values for different statistical models used to predict sensory traits in cooked sweet potatoes.

To test whether a combination of both color and GLCM texture features could be used to predict the sensory traits, a comparison of several machine learning methods in sweet potato indicated that the overall performance of the XGBoost was better in terms of *r*-squared (*r*^2^) than the other models (average *r*^2^ = 0.50) (Figure 4). This was followed by the support vector regression (NL-SVM) with radial kennel (average *r*^2^ = 0.49). The RF regressor linear regression models had the poorest performance (average *r*^2^ = 0.10–0.20). The XGBoost and NL-SVM equally had the least root mean square error (RMSE) (average = 0.92) (Figure 5) and the lowest mean absolute error (MAE) (average = 0.70 and 0.72) (not shown). XGBoost has become a popular method in image analysis, where it has outperformed many existing traditional methods in machine learning [38]. Yet SVM has become a choice method for its ability to deal with linear and nonlinear relationships; for example, in predicting sensory traits using NIR spectra, it has shown severally its superiority over other statistical methods [7]. Although we also noted that the prediction of the aromas and flavors is still high compared to other traits (except color), further investigations may be required to conclude whether these predictions are meaningful and important in practice.

For individual traits in sweet potato, excluding color that had prediction models of *r*^2^ > 0.70, moderately robust models were developed for some traits, where the maximum (max) predictions from different models were for floral flavor (*r*^2^_max_ = 0.72), pumpkin aroma (*r*^2^_max_ = 0.69), sweet potato flavor (*r*^2^_max_ = 0.66), and pumpkin flavor (*r*^2^_max_ = 0.64). Less robust models were detected for mealiness (*r*^2^_max_ = 0.50), which contrasts with the computer vision approach, which developed robust models on the same samples (*r*^2^_max_ = 0.80) using the RF regressor as the best performer [6].

**Figure 6:**
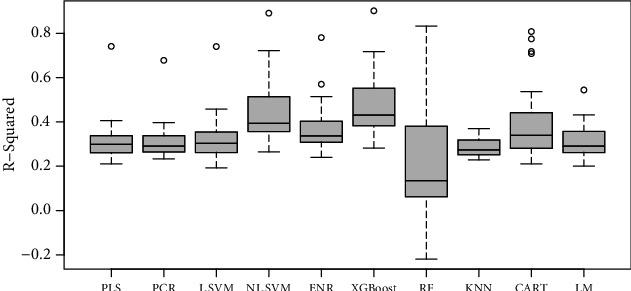
Boxplots of *r*^2^ values for different statistical models used to predict sensory traits in cooked potatoes.

**Figure 7:**
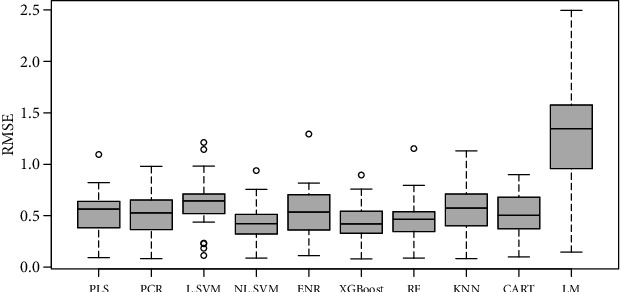
Boxplots of RMSE values for different statistical models used to predict sensory traits in cooked potatoes.

Similarly, in potato, the overall performance of the XGBoost was better in terms of *r*-squared *r*^2^ than the other models (average *r*^2^ = 0.48) (Figure 6). This was followed by the NL-SVM with radial kennel (average *r*^2^ = 0.45). The RF regressor linear regression models had the poorest performance (average *r*^2^ = 0.2). The NL-SVM (RMSE = 0.42) and XGBoost (RMSE = 0.43) had the least overall error (Figure 7). Predictions of individual traits based on color and texture features were most moderate to weak. Except for prediction models of yellow color, which reached an *r*^2^_max_ of 0.90, the highest models were developed for moisture in mass (*r*^2^_max_ = 0.78), uniformity of texture (*r*^2^_max_ = 0.72), and smoothness (*r*^2^_max_ = 0.71). Moderate prediction models were developed for potato flavor (*r*^2^_max_ = 0.57), sour taste (*r*^2^_max_ = 0.57), and bitter aftertaste (*r*^2^_max_ = 0.57).

### 3.3. GLCM Texture Features for Sweet Potato and Potato

The samples of the two crops exhibited significant differences in the texture features, with sweet potato samples exhibiting more local variations within samples than potatoes; for example, homogeneity values were higher in potatoes compared to sweet potatoes (Supporting Information Tables 4a/4b). However, the variation among the samples was lower for potatoes compared to sweet potatoes as depicted from the statistical parameters of the color and GLCM texture statistics (Supporting Information Tables 4a/4b), with implications for the modeling.

## 4. Conclusion

The results show that the instrument color prediction is sufficient for evaluating potato and sweet potato samples. Because texture is a description of how the colors change spatially, we anticipated a correlation among the features, although this was lower than expected. The grey-level arrangement of pixels (GLCM) provides a partial explanation of the sensory textural features. The protein content, but not starch, moisture, or amylose, is partially associated with the dissimilarity among the pixels. Whether color and GLCM texture parameters can predict aroma and flavor is still a question that needs further investigation. Further digital image processing techniques like smoothing, enhancement, and segmentation could also be employed to enhance the data collection from images. Future work could consider classification models based on threshold values of traits instead of correlations.

## Figures and Tables

**Table 1:** The highest correlations between the sensory and DigiEye LABCH color scales in cooked sweet potato samples. L represents brightness; for A, negative values refer to greenish, and positive values refer to red; for B, negative values refer to blue, and positive values refer to yellow; C stands for saturation; and H stands for hue.

	**Orange color intensity**	**Pumpkin aroma**	**Pumpkin flavor**	**Dry matter**
L	−0.73	−0.60	−0.61	0.50
A	0.80	0.64	0.63	−0.51
B	0.80	0.45	0.44	−.39
C	0.83	0.52	0.51	−0.44
H	−0.83	−0.66	−0.65	0.52

**Table 2:** The highest correlations between the sensory and DigiEye LABCH color scales in cooked potato samples. L represents brightness; for A, negative values refer to greenish, and positive values refer to red; for B, negative values refer to blue, and positive values refer to yellow; C stands for saturation; and H stands for hue.

	**Yellow color**	**Potato flavor**	**Bitter taste**	**Sour taste**
L	−0.26	−0.25	0.20	0.49
A	0.63	0.20	−0.28	−0.28
B	0.87	0.46	−0.52	−0.50
C	0.87	0.46	−0.52	−0.50
H	−0.76	−0.40	0.47	0.40

**Table 3:** The highest correlations between the sensory and RGB (R= red; G = green; B = blue) color scales in sweet potato cooked roots.

**Pixels**	**Orange color intensity**	**Pumpkin aroma**	**Pumpkin flavor**	**Sweet potato flavor**	**Dry matter**
Number R	0.07	0.04	0.04	0.25	0.05
Number G	−0.75	−0.62	−0.61	0.60	0.51
Number B	−0.84	−0.58	−0.57	0.49	0.47
Mean R	−0.27	−0.24	−0.26	0.34	0.26
Mean G	−0.77	−0.62	−0.62	0.58	0.51
Mean B	−0.83	−0.56	−0.56	0.49	0.46
Median R	−0.30	−0.26	−0.28	0.35	0.30
Median G	−0.77	−0.62	−0.61	0.58	0.52
Median B	−0.83	−0.58	−0.56	0.49	0.46

**Table 4:** The highest correlations (*r* > 0.50) between the sensory and GLCM traits in cooked sweet potato roots.

	**GLCM maximum mean**	**GLCM maximum variance**
Pumpkin aroma	−0.66	-0.67
Pumpkin flavor	−0.66	−0.66
Cooked carrot flavor	−0.58	−0.58
Moisture release	−0.59	−0.59

## Data Availability

The authors confirm that the data supporting the findings of this study are available within the article and its supporting information.

## Supporting Information

Supporting Information Additional supporting information can be found online in the Supporting Information section. The supporting information comprises details of the sampling sites (Supporting Information Figure 1), the list of sensory traits assessed, and their statistical descriptions (Supporting Information Table 1) plus additional correlations among color, texture, and sensory parameters. These include the highest correlations between the sensory and color scales of raw sweet potato roots shown in Supporting Information Table 2; the highest correlations between the sweet potato sensory and raw RGB color scales (Supporting Information Table 3); the statistics associated with key color and texture features in cooked sweet potato roots (Supporting Information Table 4a); and the statistics associated with key color and texture features in cooked potato roots (Supporting Information Table 4b).
