# Supplementary material for: Color and Grey-Level Co-Occurrence Matrix Analysis for Predicting Sensory and Biochemical Traits in Sweet Potato and Potato
Source: Int J Food Sci. 2024 Oct 30;2024:1350090. doi: 10.1155/2024/1350090 (PMC11617048; doi:10.1155/2024/1350090)
Supplement: Supporting Information — Additional supporting information can be found online in the Supporting Information section. The supporting information comprises details of the sampling sites (Supporting Information Figure 1), the list of sensory traits assessed, and their statistical descriptions (Supporting Information Table 1) plus additional correlations among color, texture, and sensory parameters. These include the highest correlations between the sensory and color scales of raw sweet potato roots shown in Supporting Information Table 2; the highest correlations between the sweet potato sensory and raw RGB color scales (Supporting Information Table 3); the statistics associated with key color and texture features in cooked sweet potato roots (Supporting Information Table 4a); and the statistics associated with key color and texture features in cooked potato roots (Supporting Information Table 4b). [file 1350090.f1.docx]

**Supplementary files**


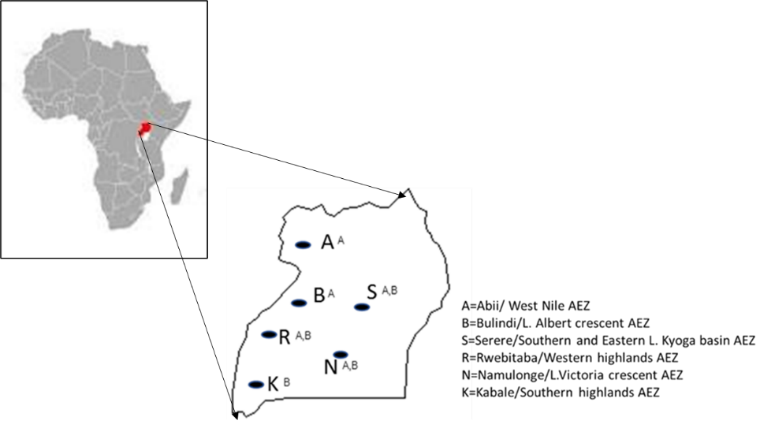


Supplementary Figure 1: Map of Uganda showing the sites where samples were collected in different agroecological zones (AEZ) for the two seasons, A and B (in superscripts). Inset is the map of Africa showing the location of Uganda (Adapted from Nantongo et al 2024b).

Supplementary Table 1: Statistical parameters of assessed sensory traits

| # | Parameter | Mean | SD | Minimum | Maximum |
| --- | --- | --- | --- | --- | --- |
|  | *Visual* |  |  |  |  |
| 1 | Orange color intensity | 3.70 | 2.99 | 0.00 | 9.18 |
| 2 | Uniformity of color | 7.12 | 1.11 | 1.75 | 9.64 |
| 3 | Degree of translucency | 1.25 | 1.24 | 0.00 | 8.33 |
| 4 | Fibrous appearance | 0.90 | 0.60 | 0.00 | 4.51 |
|  | *Aromatic* |  |  |  |  |
| 5 | Sweetpotato aroma | 5.76 | 1.31 | 1.00 | 8.10 |
| 6 | Caramel aroma | 0.56 | 1.13 | 0.00 | 6.92 |
| 7 | Pumpkin aroma | 0.37 | 0.61 | 0.00 | 3.96 |
|  | *Flavor and taste* |  |  |  |  |
| 8 | Off odour | 0.17 | 0.29 | 0.00 | 1.86 |
| 9 | Sweetpotato flavor | 5.82 | 1.45 | 0.67 | 8.30 |
| 10 | Pumpkin flavor | 0.67 | 1.27 | 0.00 | 6.83 |
| 11 | Cooked carrot flavor | 0.12 | 0.25 | 0.00 | 1.67 |
| 12 | Floral flavor | 0.11 | 0.17 | 0.00 | 1.00 |
| 13 | Sweet taste | 5.25 | 1.55 | 0.00 | 7.70 |
| 14 | Bitter taste | 0.25 | 1.05 | 0.00 | 6.42 |
|  | *Texture* |  |  |  |  |
| 15 | Hardness by hand | 4.32 | 1.73 | 0.00 | 8.78 |
| 16 | Moisture release | 0.84 | 1.31 | 0.00 | 7.25 |
| 17 | Cohesiveness | 5.75 | 2.50 | 0.17 | 9.31 |
| 18 | Crumbliness/Mealiness by hand | 4.92 | 2.33 | 0.33 | 9.25 |
| 19 | Fracturability | 3.81 | 2.06 | 0.27 | 8.25 |
| 20 | Firmness/ Hardness | 3.86 | 1.29 | 0.73 | 7.78 |
| 21 | Crunchiness | 0.60 | 0.83 | 0.00 | 5.83 |
| 22 | Moisture in mass | 4.25 | 2.20 | 0.00 | 8.90 |
| 23 | Crumbliness | 4.67 | 2.25 | 0.20 | 8.67 |
| 24 | Adhessiveness (Stickiness) | 1.53 | 0.83 | 0.11 | 6.58 |
| 25 | Fibrousness | 0.72 | 0.72 | 0.00 | 4.67 |
| 26 | Smoothness | 6.45 | 1.79 | 0.58 | 9.45 |
| 27 | Rate of breakdown | 6.13 | 1.28 | 2.13 | 8.88 |
| 28 | Dry matter | 34.96 | 7.73 | 4.50 | 46.62 |
| 29 | Peak positive force 1 | 4783.13 | 2206.69 | 1473.47 | 12709.42 |
| 30 | Peak positive force 2 | 3432.20 | 1524.10 | 1097.00 | 9779.50 |
| 31 | Positive Area 1 | 8586.60 | 3688.30 | 2716.10 | 18383.87 |
| 32 | Positive Area 2 | 3340.78 | 1664.69 | 1022.00 | 9598.63 |
| 33 | Optimal cooking time | 22.55 | 7.80 | 10.00 | 55.00 |
| 34 | Water absorption | 1.42 | 2.18 | -5.10 | 9.08 |

Supplementary Table 2: The highest correlations between the sensory and color scales of raw sweetpotato roots

|  |  | | Sweetpotato | | |
| --- | --- | --- | --- | --- | --- |
|  | Orange color intensity | Sweetpotato aroma | | Pumpkin aroma | Dry matter |
| L | -0.79 | 0.61 | | -0.65 | 0.478 |
| A | 0.82 | -0.56 | | 0.64 | -0.46 |
| B | 0.81 | -0.52 | | 0.59 | -0.41 |
| C | 0.84 | -0.56 | | 0.64 | -0.44 |
| H | -0.84 | 0.49 | | -0.60 | 0.44 |

Supplementary Table 3: The highest correlations between the sweetpotato sensory traits assessed in cooked samples and RGB color scales from raw samples

|  | Sweetpotato | | |
| --- | --- | --- | --- |
|  | Orange color intensity | Pumpkin aroma | Dry matter |
| No_Red | 0.07 | 0.04 | 0.05 |
| No_Green | -0.75 | -0.62 | 0.51 |
| No_blue | -0.84 | -0.58 | 0.47 |
| R_mean | -0.27 | -0.24 | 0.26 |
| G_mean | -0.77 | -0.62 | 0.51 |
| B_mean | -0.83 | -0.56 | 0.46 |
| R_median | -0.30 | -0.26 | 0.30 |
| G_median | -0.77 | -0.62 | 0.52 |
| B_median | -0.83 | -0.56 | 0.47 |

Supplementary Table 4a: Statistics associated with key color and texture features in cooked sweetpotato roots

|  | Mean | sd | min | max |
| --- | --- | --- | --- | --- |
| L | 64.63 | 7.94 | 50.28 | 80.31 |
| A | 14.98 | 13.50 | -2.93 | 34.73 |
| B | 40.20 | 14.54 | 8.82 | 59.09 |
| C | 44.01 | 17.22 | 8.92 | 65.09 |
| H | 75.05 | 15.35 | 54.30 | 99.68 |
| No_Red | 164.85 | 10.03 | 130.92 | 184.92 |
| No_Green | 131.56 | 24.41 | 91.07 | 181.83 |
| No_blue | 88.82 | 29.29 | 56.57 | 157.73 |
| R_mean | 0.69 | 0.04 | 0.58 | 0.75 |
| G_mean | 0.55 | 0.11 | 0.37 | 0.73 |
| B_mean | 0.37 | 0.14 | 0.21 | 0.64 |
| R_median | 0.69 | 0.04 | 0.58 | 0.76 |
| G_median | 0.55 | 0.12 | 0.37 | 0.74 |
| B_median | 0.38 | 0.14 | 0.21 | 0.65 |
| glcm_mean (min) | 0.26 | 0.15 | 0.07 | 0.73 |
| glcm_variance (min) | 101.01 | 101.09 | 4.51 | 550.36 |
| glcm_homogeneity (min) | 0.38 | 0.09 | 0.20 | 0.61 |
| glcm_contrast (min) | 0.68 | 0.69 | 0.09 | 4.38 |
| glcm_dissimilarity (min) | 0.48 | 0.27 | 0.09 | 1.49 |
| glcm_entropy (min) | 1.49 | 0.57 | 0.38 | 2.93 |
| glcm_second_moment (min) | 0.03 | 0.01 | 0.01 | 0.06 |
| glcm_mean (max) | 0.93 | 0.05 | 0.70 | 0.99 |
| glcm_variance (min) | 840.10 | 82.81 | 471.42 | 934.80 |
| glcm_homogeneity (max) | 0.78 | 0.10 | 0.50 | 0.96 |
| glcm_contrast (max) | 11.31 | 8.52 | 1.96 | 36.69 |
| glcm_dissimilarity (max) | 2.32 | 0.90 | 0.90 | 4.48 |
| glcm_entropy (max) | 3.83 | 0.29 | 3.07 | 4.29 |
| glcm_second_moment (max) | 0.37 | 0.17 | 0.09 | 0.83 |

Supplementary Table 4b: Statistics associated with key color and texture features in cooked potato roots

|  | mean | sd | min | max |
| --- | --- | --- | --- | --- |
| L | 76.61 | 3.45 | 70.66 | 84.44 |
| A | -0.29 | 1.74 | -3.75 | 2.90 |
| B | 32.69 | 9.02 | 12.66 | 45.21 |
| C | 32.75 | 8.96 | 12.89 | 45.23 |
| H | 91.49 | 4.03 | 86.17 | 100.75 |
| glcm_mean (min) | 0.25 | 0.14 | 0.04 | 0.62 |
| glcm_variance (min) | 86.61 | 89.92 | 1.85 | 387.54 |
| glcm_homogeneity (min) | 0.35 | 0.09 | 0.24 | 0.59 |
| glcm_contrast (min) | 0.85 | 0.70 | 0.10 | 3.55 |
| glcm_dissimilarity (min) | 0.58 | 0.30 | 0.10 | 1.43 |
| glcm_entropy (min) | 1.66 | 0.59 | 0.44 | 2.83 |
| glcm_second_moment (min) | 0.02 | 0.01 | 0.02 | 0.07 |
| glcm_mean (max) | 0.93 | 0.04 | 0.81 | 0.98 |
| glcm_variance (min) | 833.07 | 65.39 | 635.90 | 919.63 |
| glcm_homogeneity (max) | 0.74 | 0.11 | 0.51 | 0.95 |
| glcm_contrast (max) | 15.48 | 9.74 | 2.13 | 38.86 |
| glcm_dissimilarity (max) | 2.70 | 0.89 | 1.08 | 4.48 |
| glcm_entropy (max) | 3.96 | 0.30 | 2.97 | 4.22 |
| glcm_second_moment (max) | 0.32 | 0.18 | 0.10 | 0.80 |
